# Experimental Evaluation of the Process Performance of MF and UF Membranes for the Removal of Nanoplastics

**DOI:** 10.3390/membranes13070683

**Published:** 2023-07-21

**Authors:** Serena Molina, Helena Ocaña-Biedma, Laura Rodríguez-Sáez, Junkal Landaburu-Aguirre

**Affiliations:** 1 IMDEA Water Institute, Punto Com. nº 2, 28805 Alcalá de Henares, Madrid, Spain; 2 Chemical Engineering Department, University of Alcalá, Ctra. Madrid-Barcelona Km 33,600, 28871 Alcalá de Henares, Madrid, Spain

**Keywords:** ultrafiltration membranes, microfiltration membranes, nanoplastics, membrane process performance, BSA, synergetic effect, wastewater treatment

## Abstract

Despite the high removal ability of the wastewater treatment technologies, research efforts have been limited to the relatively large-sized microplastics, leaving nanoplastics outside the studied size spectrum. This study aims to evaluate the process performance of MF and UF membranes for the removal of single and mixed solutions of polystyrene nanospheres (120 and 500 nm) and BSA. The process performance was evaluated in terms of the rejection coefficient, the normalized flux, and the permeability recovery. The fouling mechanism of these pollutants was studied, evaluating the effect of different membrane materials, membrane pore sizes, and nanoplastic sizes, as well as the synergetic effect of the mixture of foulants. This study was complemented by surface membrane characterization. Polystyrene nanospheres were successfully removed with all the membranes studied, except for the MF membrane that obtained PS 120 nm rejection coefficients of 26%. Single nanoplastic particles were deposited in UF membranes creating a pore blocking and cake layer formation, whilst the nanoplastics of 120 nm were accumulated inside the MF membrane creating an internal pore blocking. In mixed solutions, the BSA acted in two different ways: (i) as a stabilizer, hindering the deposition of nanoplastics and (ii) as a main foulant that caused a substantial flux reduction.

## 1. Introduction

Plastics have the characteristics of being versatile, durable, cheap, and light. Therefore, they have a wide variety of applications in sectors such as medicine, construction, food, and agriculture. In fact, global plastics production increased to 390.7 million tonnes in 2021 [1]. However, due to their intrinsic properties, plastics take a long time to degrade. Consequently, there is an urgent need for efficient plastic waste management avoiding the further deterioration of this serious environmental problem. When plastics are discarded and not properly recycled, plastic waste and their fragments inevitably end up penetrating water bodies. In addition, when these plastic fragments remain in the environment for long periods of time, they break down into micro- (MPs) and nanoplastics (NPs), called secondary micronanoplastics (MNPs). MPs are defined as plastic fragments smaller than 5 mm, and NPs are defined as plastic fragments smaller than 1 μm [2]. On the other hand, primary MNPs are plastic fragments manufactured in micro–nano size, which can also be found in water bodies.

Due to their ubiquity, the concern about our exposure to them and their possible health effects has grown in the last few years. Microplastics have been detected in oceans and fresh water as well as in table salt, drinking water, and air, posing an inevitable human exposure risk [3]. Several authors [4,5,6,7] have reported that wastewater treatment plants (WWTPs) are the major sources of MNPs in freshwaters. Therefore, if they are not effectively removed from wastewater, they are discharged and reintroduced into the environment. Tang et al. [8] recently published a review article analysing numerous papers published in the last 10 years compiling the removal performance of the different stages typically found in WWTPs. In general terms, WWTPs efficiently remove MPs, obtaining removal efficiencies up to 98% and 99%, for primary and secondary treatments, respectively.

Despite the high removal ability of secondary treatment technologies such as conventional activated sludge and membrane bioreactors [9], as well as tertiary treatment technologies, such as disc filter (DF), rapid sand filtration (RSF), and dissolved air flotation (DAF) [10], the research efforts have been mainly limited to the quantification of relatively large-sized MPs, leaving small-sized MNPs outside the studied size range [11]. The evaluation of the removal capacity of small-sized MNPs by WWTPs and advanced water treatment technologies has been limited due to the lack of standardized methods for sampling, identification, and quantification of MNPs from wastewater [12]. This is an important bottleneck that hinders a real evaluation of their release into the water bodies from the WWTPs. In general terms, it is well known that WWTPs efficiently remove relatively large MPs, but small-sized MNPs can pass readily through the WWTPs entering the environment. As an example, Wolff et al. [13] identified MPs > 10 μm in a WWTP in Germany, reporting that 95% of MPs in the effluent of the secondary treatment stage were between 10 and 100 μm. Consequently, to truly understand the efficiency of wastewater treatment technologies, it is important to develop new processes capable of capturing, identifying, and quantifying the small-sized MNPs. Considering the small pore size of microfiltration (MF, 0.1–10 μm) and ultrafiltration (UF, 0.01–0.1 μm) membranes, pressure-driven membranes could not only be implemented as a tertiary treatment for the removal of MNPs but also as a sampling and preconcentration of these pollutants [11,14]. The use of MF/UF membranes in concentration mode could also improve the subsequent detection/identification steps of these pollutants, allowing reaching smaller detection and quantification limits.

In this context, it is important to conduct studies to understand the process performance of pressure-driven membranes for the separation of MNPs from water matrices, giving special attention to nanoplastics. However, very few studies have focused on the separation of nanoplastics by membrane technology. Wan et al. [15] developed nanofibrous membranes for the removal of polystyrene NPs (size between 107 and 1450 nm) by a gravity-driven membrane filtration process. They obtained 92% of NPs’ removal and observed different membrane rejection mechanisms as a function of the NPs’ size and membrane pore diameter: (i) membrane pores via the size-exclusive effect when the diameter of the NPs was larger than the membrane pore size and (ii) electrostatic attraction and hydrophobic interactions when the diameter of the NPs was smaller than the membrane pores. This aspect is related to the fouling mechanism reported by Enfrin et al. [16] using 30 kDa PES membranes with NPs’ size between 13 and 690 nm at a concentration of 10 ppm in distilled water. They proposed that fouling occurs, first, by pore blocking and, subsequently, by a cake layer formation on the surface. In addition, Pizzichetti et al. [17] studied the fouling phenomena of MF cellulose acetate membrane by polyamide (PA) and polystyrene (PS) particles in dead-end configuration. The results also showed that the prevailing mechanisms during microplastic filtrations were complete pore blocking followed by cake layer formation.

However, further studies of fouling mechanisms in membranes are required, where more realistic compositions of real water are studied in order to predict more accurately the behaviour of MNPs in water and their interaction with membranes. In particular, natural organic matter (NOM) is another pollutant that is abundant in WWTP effluents [18] and can also influence the membrane fouling mechanism. Therefore, it is essential to understand the fouling behaviour of MPs/NPs, NOM, and their combination in membrane filtration processes. NOM is a mixture of organic matter of different molecular weights and chemical properties. Regarding membrane fouling studies with NOM, the hydrophilic biopolymers (mainly proteins and polysaccharides) and the hydrophobic humic substances have been identified as the most important NOM fractions [19]. Further, BSA is widely used as a model compound of proteins for fouling studies with membranes [19,20]. Under this context, Markazi et al. [21] investigated the single and simultaneous fouling behaviour of UF PES (50 kDa) using polystyrene MPs/NPs (220 nm, 25 ppm) and bovine serum albumin (BSA, 10 mg/L). In addition, a multistage Hermia’s model was used to understand the fouling mechanism during the filtration experiments. They observed that more PS MPs/NPs were deposited on the membrane surface during the combined filtration of contaminants, with an early-stage cake layer formation.

The typical protein values for wastewaters reported in the literature are below 100 mg/L [22,23]. However, considering that the concentration ratios of the foulants might affect the fouling behaviour of membranes, further accelerated fouling studies at different concentration ratios are necessary. In addition, beside the interesting research conducted until now, most of the studies containing mixtures of NOM and MNPs have been focused on the evaluation of the fouling phenomena of one type of membrane.

The present research work goes one further step in the evaluation of the process performance of MF and UF membranes in terms of the rejection coefficient, the normalized flux, and the permeability recovery values, evaluating the separation efficiency of different nanoplastic size and mixed solutions with NPs and BSA. Further, to the knowledge of the authors, for the first time, the effect of the membrane material is also studied, complemented by the surface membrane characterization (scanning electron microscopy and confocal laser scanning microscopy).

## 2. Materials and Methods

### 2.1. Chemical Reagents and Membranes

The fluorescent polystyrene (PS, λex = 576 nm and λem = 596 nm) nanosphere latex of particle sizes of 120 nm and 500 nm were purchased from Ikerlat Polymers, Lasarte, Spain. The bovine serum albumin (MW: 67 kDa, lyophilized powder, ≥96% agarose gel electrophoresis) was purchased from Sigma-Aldrich (Spain). The flat sheet membranes used were 30 kDa Biomax polyethersulfone (PES) UF membranes and 30 kDa Ultracel regenerated cellulose (RC) UF membranes purchased from Merk-Millipore, Madrid (Spain) and chlorinated polyethylene (CPE) MF membranes with a nominal pore size of 0.4 µm from Kubota Europe, Rodgau, Germany.

### 2.2. Filtration Experiments

The filtration experiments were carried out in a solvent-resistant stirred cell (XFUF07601, Merk-Millipore) for 76 mm membranes, with a membrane effective area of 0.0040 m^2^. The filtration experiments were conducted at room temperature and a pressure of 2 bar. The applied overpressure was achieved by nitrogen gas. The stirring speed was constant to obtain effective agitation and a vortex approximately one-third of the depth of the liquid as recommended by the supplier. The initial feed volume was 200 cm^3^, and the ultrafiltration experiments were carried out until 60 cm^3^ of the total sample was filtered. Accelerated fouling experiments were conducted with synthetic feed containing PS NPs (10 ppm) and a mixture of BSA (1 g/L) and PS NPs (10 ppm) of two different particle sizes (120 and 500 nm).

The process performance was evaluated in terms of the rejection coefficient (*R*, %), calculated with Equation (1), and the normalized flux (*Jv*/*Jw*). The normalized flux was calculated by dividing the permeate flux of the solution by the clean water flux obtained with the pristine membrane. The permeate flux was calculated by measuring the time required for every 20 mL of permeate collected following Equation (2).

(1) $$R\;\left(\%\right)=(1-\frac{Cp}{Cf})\times 100,$$

where *Cp* and *Cf* are the concentrations of the NPs or BSA in the permeate and the feed site, respectively. The NPs and BSA concentration in the feed and in the permeate were quantified by UV-vis spectrophotometry (Shimadzu UV-1800 model) at a wavelength of 574 nm and 278 nm for PS and BSA, respectively.

(2) $$J=\frac{V}{A\times ∆t},$$

where *V* (L) is the volume of the permeated water, *A* (m^2^) is the membrane area, and Δ*t* is the permeation time.

After conducting the filtration experiments with the studied solutions, the membrane was cleaned, first, by rinsing it with Milli-Q water and then by conducting another filtration with Milli-Q water. The water permeability of the membranes before the filtration experiment using NPs (*Lp*_1_) and after the cleaning step (*Lp*_2_) was calculated in order to evaluate the permeability recovery (*PR*, %) of the membrane as follows,

(3) $$PR\;\left(\%\right)=\frac{Lp1}{Lp2}\times 100.$$

In addition, the water permeability of the membranes was calculated following Equation (4), where the permeability is the slope of the linear equation obtained when representing the water flux against the transmembrane pressure.

(4) Jw=Lp×∆P.

### 2.3. NPs and Membranes Characterization

#### 2.3.1. Dynamic Light Scattering (DLS) and ζ-Potential

The size and surface charge of the PS NPs in the synthetic feed solutions were determined by dynamic light scattering and the ζ-potential analyser (Zetasizer Nano ZSE, Malvern Paranalytical, UK) with a refractive index of 1.59 and an absorbance of 0.01 at pH 7. For the determination of the wet particle diameter, the Hydro SM dispersion unit was selected, using water as the dispersant and a parameter refractive index (IR) of 1.33, an obscuration between 5 and 15%, a recirculation speed of 2500 rpm, and a measurement time of 30 s. The analysis of the results was conducted using the Mie scattering model, which achieved the quality levels required by ISO 13320:2020 (Particle size analysis: laser diffraction methods).

Measurements of the mixed solutions of nanospheres and BSA were conducted to determine whether the presence/absence of BSA affected the particle diameter of the studied PS nanospheres due to the formation of aggregates. Table 1 shows the average size values (D, volumetric particle diameter, nm) of the PS nanospheres and BSA, the polydispersity (PDI), and the ζ-potential values.

The measured diameters of the nanospheres were very similar to the size specified by the manufacturer. In addition, there was no apparent difference between the diameter obtained for PS NPs solution and the mixed solutions of PS NPs with BSA. Consequently, there were no aggregates in either of the solutions studied that could influence the rejection coefficients obtained during membrane filtration experiments. On the other hand, the small polydispersity index (PDI < 0.2) also indicated the monodispersity of all the solutions studied. This result is in concordance with the data recently reported by Wan et al. [15].

Regarding the ζ-potential values measured at pH 7, similar values were obtained for the solutions of the single PS 500 nm nanosphere and the mixed solution with BSA. Further, the ζ-potential of the PS 120 nm nanosphere slightly decreased in the presence of BSA. Similar ζ-potential values (−39.4 ± 3.9 mV) were obtained by Miao et al. [26] for polystyrene beads of approximately 130 nm in Milli-Q. However, Wan et al. [15] reported ζ-potential values between −5 and −25 mV in PS beads of different diameters for the same pH values. Wan et al. analysed non-functionalized PS beads. In the present work, the NPs used were functionalized with carboxyl groups, which explains the more negative zeta potential values obtained.

#### 2.3.2. Scanning Electron Microscopy (SEM) and Confocal Laser Scanning Microscopy (CLSM)

The morphological properties of the membrane surfaces and the distribution of NPs in the membrane surfaces were examined by scanning electron spectroscopy (SEM) (S-8000 Model (Hitachi, Tokyo, Japan)). All the analysed membranes were previously dried at 50 °C for 48 h. A confocal laser scanning microscope (CLSM Leica SP5, Leica Microsystems, Wetzlar, Germany) was used to complement the information obtained by SEM. Two different areas (5 mm × 5 mm) of each membrane were analysed. The membrane images were analysed by the ImageJ software. The 3D projection images of the NPs were constructed by ImageJ 3D viewer plugin, overlaying different images of the CLSM. The overlayed images were taken every 1 ym in depth from the fouling surface to the inside of the membrane.

**Figure 1:**
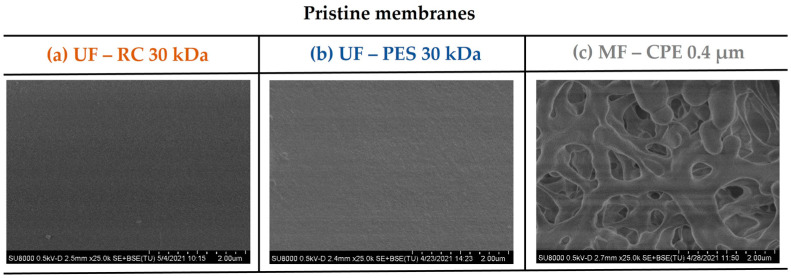
SEM images of the pristine membrane surfaces. (**a**) UF—RC 30 kDa; (**b**) UF—PES 30 kDa; (**c**) MF—CPE 0.4 μm.

Figure 1 shows the surface SEM micrographs of the pristine membranes studied. As Table 2 shows, the roughness values (Ra and Rq) obtained for the UF membranes were two orders of magnitude lower than the MF membrane, reported previously by Rodriguez-Sáez et al. [27]. Other significant differences between the three membranes studied were the hydrophobic character (contact angle) and the zeta potential of the membranes, which influenced the fouling behaviour of the membranes.

## 3. Results and Discussion

### 3.1. Rejection Coefficients

Table 3 shows the rejection coefficients of the PS nanospheres and the BSA obtained with the studied UF and MF membranes. The pore size of the membranes studied determined the membrane separation efficiency, due to the steric exclusion of the compounds studied. As Table 2 shows, the UF membranes had an MWCO smaller than the size of the studied nanoplastics (120 nm and 500 nm) and BSA (66 kDa). Consequently, and as expected, both UF membranes showed excellent rejection coefficients towards the PS nanoplastics and BSA due to the molecular sieving mechanism. However, as reported by Rohani and Zydney [33], electrostatic interactions have also been demonstrated to play a key role in the separation mechanism of compounds such as proteins by ultrafiltration membranes. In this context, it is interesting to note that the RC membrane studied was slightly negative for pH > 3, with a ζ-potential value near −2 mV at pH 7, whilst the PES membrane had a much higher negative ζ-potential value (−15.7 mV). Considering that the BSA was also negatively charged, the electrostatic repulsion forces between the PES membrane and BSA were much higher than the RC and BSA. Consequently, the BSA rejection coefficient obtained with the PES membrane was higher than with the RC membrane.

On the other hand, the rejection coefficients obtained with the MF membranes varied depending on the solutions and compounds filtered. Considering that the MF membrane studied had a nominal pore size of 400 nm, the results obtained were in line with the molecular sieving mechanism, obtaining rejection coefficients of 100% for the PS of 500 nm, whilst the PS of 120 nm were not retained by the MF membranes. As expected, the MF membranes showed no rejection capacity towards the BSA because the nominal pore size of the MF membrane was much larger that the BSA molecule. However, in the presence of the PS nanospheres of 500 nm, the rejection coefficient of the BSA significantly increased obtaining a rejection coefficient of 86.33%, which showed a clear interaction between these two compounds that enhanced the separation efficiency of the MF membranes towards the BSA. Previous studies reported that it is plausible to have PS MPs/NPs partially covered by BSA molecules [21].

### 3.2. Permeate Flux and Fouling Phenomena of Single Compounds

**Figure 2:**
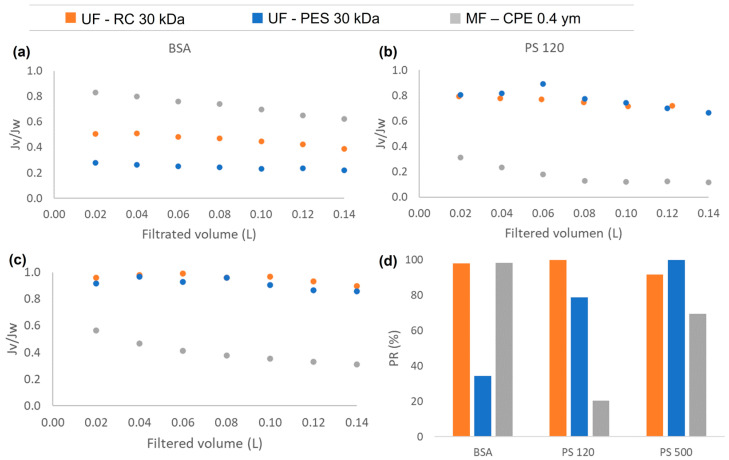
The normalized permeate flux (*Jv*/*Jw*) obtained during the filtration experiments of single compounds: (**a**) BSA, (**b**) PS 120, (**c**) PS500; and (**d**) the permeability recovery of the three studied membranes for each single compound.

Regarding the permeate flux, Figure 2 shows the normalized permeate flux obtained during the filtration experiments of the single compounds (BSA; PS 120 and PS 500) with the studied membranes. As can be observed from Figure 2a, the UF membranes showed a larger flux reduction than the MF membrane with the BSA solution. When studying the protein fouling, the ratio of the protein size to the membrane pore size and the protein–protein and protein–membrane interactions are the main roles determining the fouling degree [34]. In the case of the UF membranes, both membranes rejected the BSA protein completely, causing an accumulation of the protein on the membrane surface and, consequently, forming a homogeneous fouling layer [21]. It is important to note that the PES membrane showed a more pronounced flux reduction than the RC membrane. This is most probably due to the greater hydrophobic character of the PES membrane. When the hydrophobic–hydrophobic interaction between the BSA and the membrane was stronger, the adsorption of the BSA on the membrane surface was enhanced; consequently, a larger fouling layer was formed on the membrane surface. This also explains the lower permeability recovery values obtained with the PES membranes than with the RC membranes (Figure 2d). In the case of the RC membranes, the BSA–membrane hydrophobic interaction was weak, which means that the fouling layer deposited on the membrane was reversible. Consequently, after cleaning the membrane with MiliQ water, the BSA was successfully removed obtaining very high permeability recovery values (98%). However, in the case of strong hydrophobic interaction between the PES and BSA, the BSA adsorption is strong and irreversible, which means that the BSA was not successfully removed after the cleaning process, recovering only the 38% of the original water permeability value. In the case of the MF membrane, the BSA protein was not totally rejected and passed though the MF membrane, which might have caused a slight adsorption of the BSA inside the membrane pores that could efficiently be removed, obtaining very satisfactory permeability recovery values (98.3%).

Regarding the PS nanospheres of 120 and 500 nm (Figure 2b,c), the experiments conducted with the UF membranes showed very similar flux behaviour, where the size of the nanosphere clearly affected the membrane process performance in terms of the flux reduction. For both UF membranes, the PS of 120 nm showed a smaller normalized flux, obtaining a reduction of 35% and 25% for the PES and RC membranes, respectively. In the case of the 500 nm PS nanospheres, the normalized flux was reduced only by 15% and 10% for the PES and RC membranes, respectively. The PS nanospheres of 120 and 500 nm were completely rejected by the UF membranes, which caused the deposition of the nanospheres on the membrane surface, blocking the pores, and forming a cake layer [21]. However, larger particles form looser and more porous cake layers than smaller particles, and as a consequence, membrane fouling is less pronounced than with smaller particles [35]. In addition, it has to be mentioned that with the same feed concentration of nanoplastics (10 ppm), there was a higher number of 120 nm PS nanospheres than 500 nm PS nanospheres present in the latex solution. This might have led to a stronger interaction between the 120 nm PS nanospheres and the membranes, which might have also enhanced the accumulation of the 120 nm PS nanospheres on the membrane surface [36]. Nevertheless, the permeability recovery values obtained for both UF membranes after filtering PS of 120 and 500 nm nanospheres were high (Figure 2d). This shows that the deposition of the nanospheres on the membrane surface was reversible.

**Figure 3:**
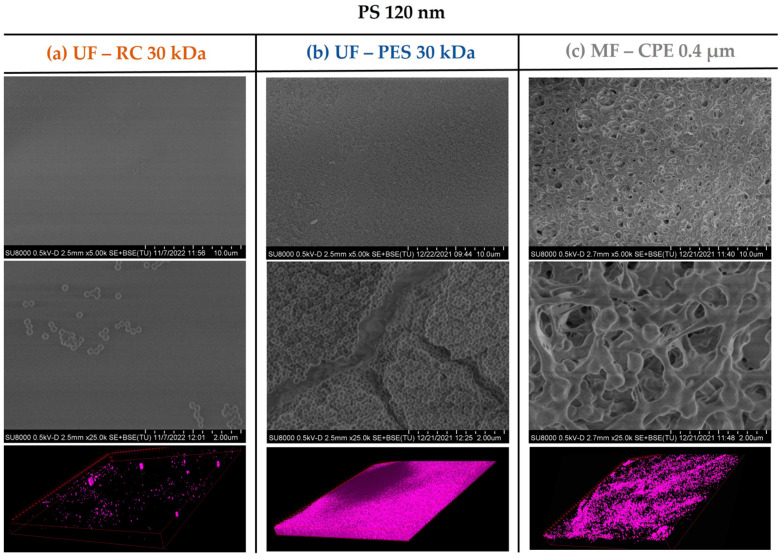
SEM images of the membrane surfaces fouled with PS 120 nanospheres and 3D-projection CLSM images of the PS 120 constructed from the fouling surface to the inside of the membranes. The pink colour represents the fluorescent PS nanospheres. Column (**a**) UF—RC 30 kDa; Column (**b**) UF—PES 30 kDa; Column (**c**) MF—CPE 0.4 μm.

**Figure 4:**
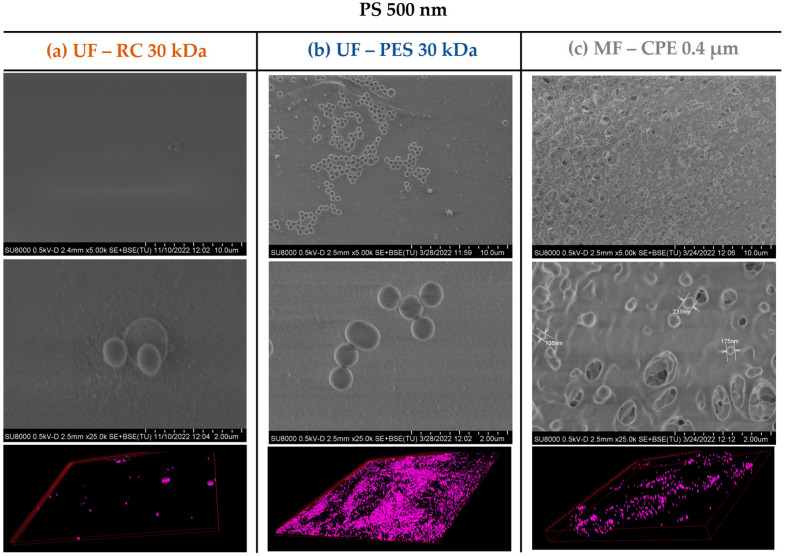
SEM images of the membrane surfaces fouled with PS 500 and 3D-projection CLSM images of the PS 500 constructed from the fouling surface to the inside of the membranes. The pink colour represents the fluorescent PS nanospheres. Column (**a**) UF—RC 30 kDa; Column (**b**) UF—PES 30 kDa; Column (**c**) MF—CPE 0.4 μm.

The fouling mechanism and the degree of membrane fouling was also corroborated with the membrane characterization images obtained by SEM and CLSM. As Figure 3 and Figure 4 show, the UF membranes showed a greater deposition of the 120 nm nanospheres on the membrane surface than the 500 nm PS nanospheres. In addition, it is important to note that the fouling degree between the two UF membranes studied (PES and CR) was very different even though both had the same MWCO. As the SEM and CLSM images in Figure 3 show, the PS 120 nm generated a thick cake layer on the membrane surface, whilst the RC membrane showed a significantly lower PS 120 nm nanosphere deposition on the membrane surface. This difference was due to the different material and hydrophilicity of both membranes. It is well known that PES membranes are more hydrophobic than RC membranes (Table 2), which causes a stronger interaction with hydrophobic foulants. The more hydrophobic character of the PES membrane enhances the adsorption of the particles on the membrane surface [16], which ultimately forms a thick cake layer, as in the case of the PS 120 nm nanospheres. On the contrary, RC membranes are more hydrophilic membranes, where the hydrophobic interactions between the RC membrane and PS nanospheres are weak. Consequently, the deposition of the nanospheres on the membrane surface was significantly lower.

In the case of the MF membrane, the fouling behaviour differed from the UF membranes mainly due to the relation of the membrane pore size and the size of the PS nanospheres. As shown in Table 3, 120 nm PS nanospheres were not retained and passed through the MF membrane. This was in concordance with the SEM images of Figure 3, where no deposition of 120 nm PS nanosphere on the membrane surface was observed. However, the CLSM images clearly showed an accumulation of the PS 120 nm in the membrane, which might be due to the retention of the 120 nm PS nanospheres inside the membrane porous structure, causing internal pore blocking of the MF membranes. This is in concordance with previous works, where it was reported that the fouling by small particles within the critical range size (when the size of the foulant is 1/6–1/2 of the pore diameter) would block the internal membrane pores, creating internal fouling [37]. In addition, it is important to note that the internal fouling was mainly irreversible, achieving very low permeability recovery values (21%).

On the other hand, in the case of the 500 nm PS nanospheres, the SEM images (Figure 4) showed a slight deposition of the 500 nm PS nanospheres on the membrane surface, which was expected considering that the 500 nm PS nanospheres were larger than the pore size of the MF membrane (average nominal size of 400 nm). In addition, the CLSM images clearly showed a fouling layer formed even though the accumulation of the 500 nm PS was clearly less pronounced than the accumulation of the 120 nm PS nanospheres. As in the case of UF membranes, where the 500 nm PS nanospheres were also deposited on top of the membrane, the permeability recovery values were successful, showing once again the reversible character of the fouling layer.

### 3.3. Permeate Flux and Synergetic Fouling Phenomena of Mixtures

**Figure 5:**
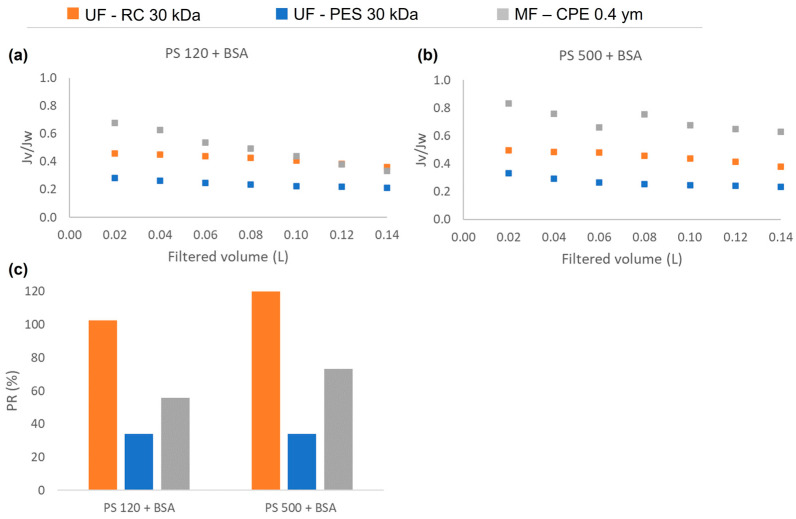
The normalized permeate flux (*Jv/Jw*) obtained during the filtration experiments of mixed solutions: (**a**) PS120 + BSA, (**b**) PS500 + BSA; and (**c**) the permeability recovery of the three studied membranes for each mixed solution.

As Figure 5 shows, in the case of having mixed solutions of BSA and NPs, the normalized flux obtained with the UF membranes was lower than with the MF membranes. Furthermore, the normalized flux obtained with both UF membranes behaved similarly to the normalized flux obtained with single solutions of BSA (Figure 2, Section 3.2). This shows that in mixed solutions that contain BSA and PS nanosphere, the protein is the main foulant affecting the flux reduction. Further, in concordance with the results obtained in Section 3.2, the hydrophobic PES membranes obtained much lower permeability recovery values than the hydrophilic regenerated cellulose membranes. This is due to a strong hydrophobic–hydrophobic interaction between the BSA and the membrane, which enhanced the adsorption of the BSA on the membrane surface. For MF membranes, the permeability recovery values were higher for the PS 500 + BSA mixture than for the PS 120 + BSA mixture. In the case of the PS 120 + BSA, both compounds readily passed through the membrane, which might have caused irreversible internal pore blocking and consequently lower permeability recovery values. However, it has to be noted that the normalized flux of mixed solution (Figure 5) of the MF membrane was higher than the normalized flux obtained with the single PS solutions (Figure 2), showing that the interaction of BSA and the nanosphere had a direct influence on the membrane fouling mechanism.

**Figure 6:**
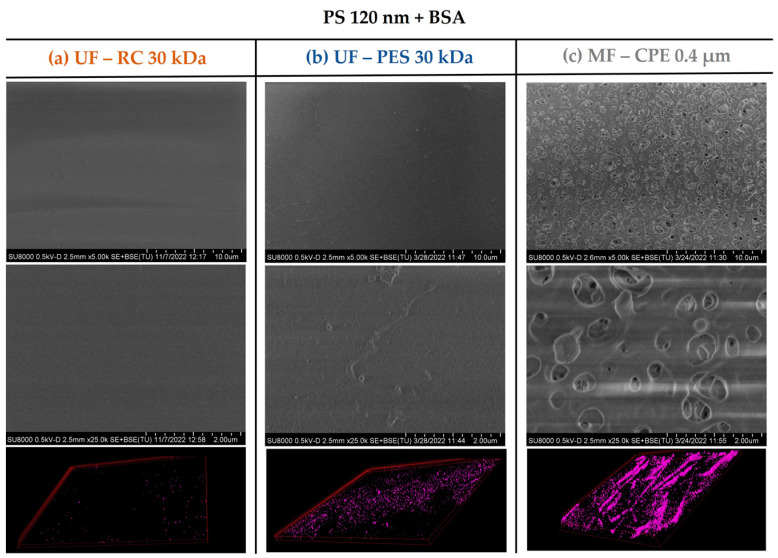
SEM and 3D-projection CLSM images of the membranes studied when filtering the PS 120 + BSA mixed solution. The pink colour represents the fluorescent PS nanospheres. Column (**a**) UF—RC 30 kDa; Column (**b**) UF—PES 30 kDa; Column (**c**) MF—CPE 0.4 μm.

**Figure 7:**
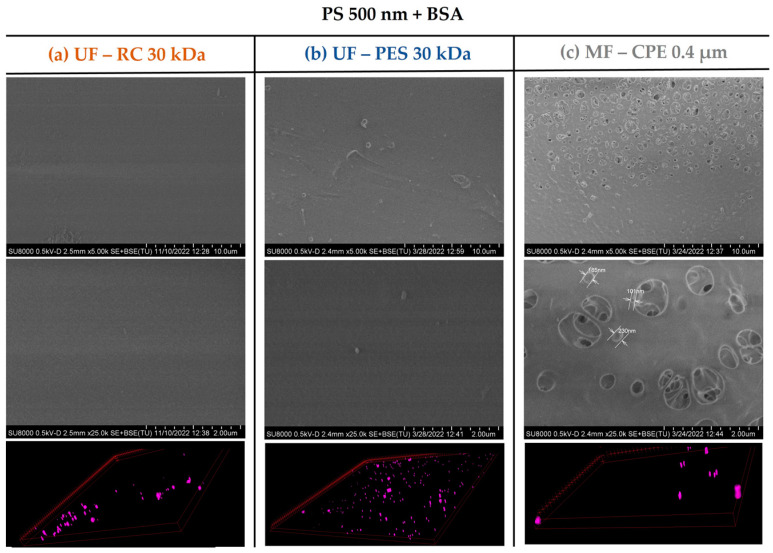
SEM and 3D-projection CLSM images of the membranes studied when filtering the PS 500 + BSA mixed solution. The pink colour represents the fluorescent PS nanospheres. Column (**a**) UF—RC 30 kDa; Column (**b**) UF—PES 30 kDa; Column (**c**) MF—CPE 0.4 μm.

This was also verified with the surface characterization of the studied membranes by the SEM and CLSM images. As Figure 6 and Figure 7 show, in both mixed solutions studied, the accumulation of the PS nanosphere on the membrane surface of the UF membranes was not very pronounced. It is important to note that in Section 3.2, Figure 3, the PES membrane showed a clear thick cake layer formation that in the case of the PS + BSA mixtures was not observed. This might be due to the synergetic effect of BSA and PS that allowed a better dispersion of the nanospheres on the bulk solution, hindering the accumulation of the NPs on the membrane surface.

Markazi et al. [21] also concluded that PS MPs/NPs as a single pollutant have a different fouling mechanism and attraction to the PES UF membrane surface in the presence of other contaminants such as BSA. However, contrary to the results of this work, they observed that more PS MPs/NPs nanoparticles were accumulated on the membrane surface in the presence of BSA. This difference might be mainly due to the concentration ratio of the PS and BSA. In the case of Markazi et al., the BSA concentration (10 mg/L) studied was lower than the concentration of the PS NP/MPs (25 mg/L), whilst in the present work, the BSA concentration (1 g/L) was much higher than the concentration of the NPs (10 mg/L). As expected, the concentration of the foulants and the ratios among different foulants affects the synergetic effect on the fouling mechanism. The research focused on mixed solutions of NPs and other compounds such as NOM is still in its infancy, and further studies in this matter are required.

## 4. Conclusions

Plastics, once in the environment, undergo abiotic and biotic weathering processes that cause their degradation and fragmentation into smaller particles: microplastics (MPs < 5 mm) and nanoplastics (NPs < 1 µm). Wastewater treatment plants (WWTPs) have been identified as one of the dominant sources of MPs in freshwater. Despite the high removal ability of the wastewater treatment technologies, research efforts have been limited to the relatively large-sized MPs, leaving NPs out of the studied size spectrum. To the knowledge of the authors, this study represents the first, where the process performance of MF and UF membranes was evaluated for the removal of nanoplastics. For this purpose, a chlorinated polyethylene MF membrane (pore size: 0.4 µm) and two different UF membranes (regenerated cellulose and polyethersulfone with MWCO: 30 kDa) were used for the removal of single and mixed solutions of polystyrene nanospheres (120 and 500 nm) and BSA. In order to understand the fouling mechanism of the studied membranes, the surface characterization of the membranes was conducted using SEM and CLSM images. This study shows that the use of CLSM images was of special interest to obtain a better understanding and comparison of the PS nanosphere deposition on/into the different membranes studied. The main findings of the study are summarized as follows:

- All the membranes studied showed successful removal towards the single solutions of the 120 nm and 500 nm PS nanosphere, except for the MF membrane that showed a very low rejection coefficient of PS 120 nm that passed the membrane readily.
- NPs that were successfully rejected by the membranes were deposited on the membrane surface generating pore blocking and/or cake layer formation. However, the permeability recovery values were very successful. It was concluded that even for the PES membrane, where a thick cake layer was observed, the fouling was reversible.
- For the removal of the 120 nm PS nanospheres, the MF membranes obtained very low permeability recoveries due to the irreversible internal pore blocking caused by the partial retention of the NPs inside the membrane pores.
- This study shows that the membrane material has a direct effect on the membrane fouling. The PES membranes have a higher hydrophobic character, which enhances the hydrophobic–hydrophobic interaction between the foulants and the membrane.
- The mixed solutions helped to understand the synergetic effect of PS NPs and BSA. It was concluded that the BSA acted in two different ways: (i) as a stabilizer that helped to have a better dispersion of NPs, which hindered the pore blocking and the cake layer formation of the PS NPs and (ii) as the main foulant that showed the highest contribution to the normalized flux reduction, decreasing the permeability recovery factor.

The evaluation of pressure-driven membranes for the removal of nanoplastics in mixed solutions is in its infancy. To better understand the synergetic effect of different compounds and the effect of the concentration ratios on the membrane process performance, further research should be focused on conducting experiments with water matrices. Special attention should be taken in the use of NP mixtures of different polymers and other compounds such NOM that can be found in the WWTPs and the environment.

## Figures and Tables

**Table 1:** Volumetric particle diameter, PDI, and ζ-potential of the PS nanospheres.

Sample	D (nm)	PDI	ζ-Potential (mV)
BSA	3 [24]	-	−15.7 [25]
PS 120 nm	122.6 ± 0.7	0.029	−45.5 ± 0.3
PS 500 nm	517.3 ± 2.9	0.048	−40.5 ± 0.1
PS 120 nm + BSA	128.1 ± 1.1	0.050	−38.3 ± 0.2
PS 500 nm + BSA	517.8 ± 2.3	0.053	−41.5 ± 0.8

**Table 2:** Technical data of the analysed membranes in this study.

Membrane	Material	Nominal Pore Size	Ra (nm)	Rq (nm)	Contact Angle	Zeta Potential (mV)
UF—RC 30 kDa	Regenerated cellulose	<15 nm	6.7 ± 1.9	8.4 ± 2.3	26 ± 3.0° [28]	−2.0 [29]
UF—PES 30 kDa	Polyethersulfone	<15 nm	7.0 ± 1.5	8.8 ± 1.9	67.6 ± 3.0° [16]	−15.1 ± 0.8 [30]
MF—CPE 0.4 μm	Chlorinated polyethylene	0.4 µm	184 ± 21 [27]	234 ± 26 [27]	104° [31]	−60.5 ± 0.7 [32]

**Table 3:** Rejection coefficients of PS NPs, BSA, and the mixed solutions.

Membrane Identification	Solution Description		PS Rejection (%)	BSA Rejection (%)
Material	Name	Size		
UF—RC 30 kDa	PS 120	120 nm	100	-
	PS 500	500 nm	100	-
	BSA	66 kDa	-	91.61
	PS 120 + BSA	Mixture	100	100
	PS 500 + BSA	Mixture	100	100
UF—PES 30 kDa	PS 120	120 nm	100	-
	PS 500	500 nm	100	-
	BSA	66 kDa	-	96.79
	PS 120 + BSA	Mixture	100	100
	PS 500 + BSA	Mixture	100	100
MF—CPE 0.4 μm	PS 120	120 nm	26.72	-
	PS 500	500 nm	100	-
	BSA	66 kDa	-	0.76
	PS 120 + BSA	Mixture	0	3
	PS 500 + BSA	Mixture	100	86.33

## Data Availability

Not applicable.
